# A Cross-Sectional Analysis Investigating Pregnant Women’s Renal Function and Its Association with Lead and Cadmium Exposures—The DSAN Birth Cohort Study in Recôncavo Baiano, Brazil

**DOI:** 10.3390/toxics12040261

**Published:** 2024-03-30

**Authors:** Eréndira C. Di Giuseppe, Homègnon A. Ferréol Bah, Erival A. Gomes Júnior, Nathália R. dos Santos, Daisy O. Costa, Victor O. Martinez, Elis Macêdo Pires, João V. Araújo Santana, Filipe da S. Cerqueira, José A. Menezes-Filho

**Affiliations:** 1 Icahn School of Medicine at Mount Sinai, New York, NY 10029, USA; edigiuseppe@berkeley.edu; 2 Institute of Collective Health, Federal University of Bahia, Salvador 40170-115, Brazil; ferreol88@gmail.com; 3 Graduate Program in Food Science, College of Pharmacy, Federal University of Bahia, Salvador 40170-115, Brazil; erivaljr@hotmail.com; 4 Graduate Program in Pharmacy, College of Pharmacy, Federal University of Bahia, Salvador 40170-115, Brazil; nathalia-rib@hotmail.com (N.R.d.S.); oliveira_daisy@hotmail.com (D.O.C.); victor_otero1@hotmail.com (V.O.M.); 5 Laboratory of Toxicology, College of Pharmacy, Federal University of Bahia, Salvador 40170-115, Brazil; elismp2014@gmail.com (E.M.P.); joao.v.santana@hotmail.com (J.V.A.S.); cerqueira.filipe@ufba.br (F.d.S.C.)

**Keywords:** kidney dysfunction, toxic metals, pregnancy, social inequities, lead, cadmium, alcohol, eGFR, oral contraceptives

## Abstract

Kidney dysfunction is increasing worldwide and is exacerbated by exposure to toxic metals. Also, pregnancy poses an overload on kidney function. We investigated how blood lead (PbB) and cadmium (CdB) levels were associated with kidney function in pregnant women from Recôncavo Baiano, Brazil, during their second trimester. In this cross-sectional study, the estimated glomerular filtration rate (eGFR) was calculated from serum creatinine and whole blood metal levels were measured by graphite furnace atomic absorption spectrophotometry in 136 volunteers. Sociodemographic data were collected using semi-structured questionnaires. The medians (IQR) of PbB, CdB, and eGFR were 0.85 µg/dL (0.45–1.75), 0.55 µg/L (0.08–0.91), and 121.8 mL/min/1.73 m^2^ (106.0–127.9), respectively. PbB medians were significantly higher in the eGFR < 90 group at 2.00 µg/dL (0.83, 3.10). After age-adjusted logistic regression, pregnant women with elevated PbB levels had decreased eGFR (OR = 1.82; 95%-CI, 1.14–3.14). However, the participants with elevated PbB levels who reported consuming alcohol during pregnancy or had CdB in the highest tertile had higher odds of reduced eGFR (OR = 2.44; 95%-CI, 1.30–5.47) and (OR = 11.22; 95% CI, 2.53–103.51), respectively. These results suggest that low Pb exposure may affect kidney function in pregnant women and calls for further investigation into toxic metal co-exposures on kidney function during pregnancy in at-risk communities.

## 1. Introduction

Kidney function is essential for processing xenobiotics and maintaining overall health, especially during pregnancy. During the gestational period, the kidneys undergo taxing physiological changes in response to the hormonal evolution in pregnant women and their environment [1]. Worldwide, and particularly in low–middle income communities, kidney dysfunction has increasingly burdened the health-care system, is a risk factor for chronic diseases, adverse pregnancy outcomes, and its prevalence has been exacerbated by exposure to toxic metals [2,3,4]. Few studies explore low-level toxic metal exposure and the kidney function of pregnant women and even fewer studies in developing countries, where inequities favor broader exposure opportunities.

Exposure to Pb and Cd at any concentration results in accumulation in many tissues. Before arriving to the kidneys, inhaled or ingested Pb is initially accumulated in erythrocytes [5]. Effects of Pb in the kidneys include impairment of the proximal tubular architecture, histological changes, mitochondrial swelling, glomerulosclerosis, and interstitial fibrosis [4,6]. When Cd is ingested, it is transported to the liver and kidneys after forming a complex with metallothionein [4,7]. Cd-metallothionein complex is readily reabsorbed by proximal tubular cells due to its low molecular weight. There, signs of cell apoptosis, membrane rupture of the proximal tubular cells of the nephron, and cytokine pathway activation leading to chronic oxidative stress are common [4,8]. These effects alone and combined are risk factors for developing hypertension, cardiovascular disease, chronic kidney disease, and adverse pregnancy outcomes [6,8,9].

Alone, pregnancy is strenuous on the kidneys and imposes histological changes to accommodate increased blood flow to the growing fetus and pregnant person [1,10,11]. Should the kidneys not maintain the necessary changes, such as an increase in eGFR, spontaneous abortion has been recorded [1]. Furthermore, studies have described associations with preeclampsia in pregnant people with lead and cadmium exposures [12,13] and the development of kidney disease in the general population and in children with prenatal non-essential metal exposures [14,15]. Few studies have focused on the deleterious outcomes of toxic metals on the pregnant person’s kidney health instead of pregnancy outcomes [16,17,18].

Exposures to Pb and Cd are pervasive worldwide due to industrialization, but low socioeconomic status is a noteworthy risk factor for both exposure and diseases that increase susceptibility to the effects of toxic metal exposure [7,19]. Like other developed countries, factors independently associated with increased Pb and Cd exposure in the USA include age, racial/ethnic minorities, low-income individuals in disadvantaged neighborhoods, and birthplace outside of the USA [19,20,21,22]. In Brazil, the primary factors significantly associated with increased Pb and Cd exposure were cigarette smoking, contaminated drinking water, proximity to industrial processes, and diet [7,23]. For pregnant women within Recôncavo Baiano, Brazil, Bah et al. (2020, 2023) found that low socioeconomic status, domestic waste burning, passive smoking, multiparity, and housing renovations were significantly associated with having higher levels of Pb and Cd [24,25]. Exposure to these toxic metals is particularly recurrent in Aratuípe and Nazaré, municipalities of Recôncavo Baiano, because of the residents’ geographic location near estuarian rivers and a primarily shellfish diet. Shellfish are reservoirs for contaminants since they are filter feeders and toxic metals accumulate in sediments and water [26,27]. Furthermore, food in this region is commonly prepared using Pb-glazed pottery produced in Maragogipinho Village in Aratuípe and sold in Nazaré and elsewhere [24,25].

To date, the only studies that studied the interaction of low-level non-essential toxic metal exposure and kidney function in pregnant people were conducted in Massachusetts, USA, Martapura, Indonesia, and Suriname [16,17,18]. To address this research gap, our study examined low-level exposure to Pb and Cd, potential socioeconomic and environmental risk factors, and kidney function in second trimester pregnant women from Recôncavo Baiano, Brazil. We hypothesized that increased exposure to Pb and Cd would be jointly associated with reduced eGFR and that it would be more pronounced among those with existing comorbidities, domestic waste burning, housing renovation, water treatment, and with a history of smoking.

## 2. Materials and Methods

### 2.1. Study Population and Design

This is a sub-analysis of the birth cohort study “*Socioenvironmental Determinants of Child Neurodevelopment*” (*DSAN-12M*). The pregnant women recruited into the DSAN-12M study are from the Nazaré das Farinhas and Aratuípe municipalities in the Recôncavo Baiano, Brazil. Detailed information on this study’s demographic characteristics, recruitment strategy, and duration has been described elsewhere [24,25]. In this study, we investigated the relationship between Pb and Cd exposures and measures of kidney function in pregnant women by using biospecimen and semi-structured survey data.

The DSAN-12M study leveraged the existing National Health System’s network of 11 primary care units (PCU) in the municipalities of Nazaré and 4 in Aratuípe, which provide direct prenatal health care services. During the prenatal consultations, pregnant women were briefly introduced to the project by the attending nurse and were invited to receive research staff at their homes for more information on the study goals and methods. Once the study details were discussed, a signed consent form was collected from consenting adults or the legal guardians of pregnant adolescents. Data collected from July 2019 to March 2020 and from July 2021 to September 2022 included, but were not limited to, surveys and blood specimens [28,29].

The primary inclusion criteria were pregnant women of a gestational age less than 24 weeks who had their first prenatal consultation at one of the PCUs and had lived in the region for at least one year before pregnancy. Exclusion criteria were women with twin pregnancies, who were prescribed medications that were potentially neurotoxic for the fetus, who were classified as high-risk pregnancies, or had a history of complicated pregnancies. Of the 164 pregnant women enrolled in the DSAN-12M study, this analysis considered the 136 participants who had sufficient whole blood sample to quantify Pb, Cd, and serum for creatinine determination for GFR estimation.

### 2.2. Sociodemographic Data

Semi-structured surveys were conducted by trained staff that collected sociodemographics and the pregnant women’s lifestyle, health status, education level, occupation, and probable sources of metal exposure. Sources of toxic metal exposure included waste disposal, water treatment, active or passive smoking, living near main roads, and household renovation. Household income level was dichotomized into “Up to 1 salary” or “Above 1 salary” according to the Brazilian minimum wage as of 1 January 2023 (Minimum wage in Brazil, Agência Brasil). Socioeconomic status (SES) data were collected based on the five categories set by the Brazilian Association of Population Studies but were dichotomized during analysis into (B/C) and (D/E) due to study population characteristics (“Critério Brasil—ABEP,” 2023). Women were considered passive smokers if they answered “yes” to “Do you live with someone who smokes?”.

### 2.3. Metal Exposure Assessment

Detailed explanation of biological data collection and processing were previously described [25,28]. Briefly, a whole blood specimen was processed and analyzed by graphite furnace atomic absorption spectrophotometry (GFAAS) to determine Pb and Cd concentrations according to Menezes-Filho et al. and Kummrow [29,30]. Quality assurance for PbB was conducted by analyzing blood samples from the Proficiency Program for Blood Lead Analysis at Instituto Adolfo Lutz (IAL, São Paulo, Brazil) concomitantly in every run. The precision and accuracy obtained were 8.8% and 107.4%, respectively. The method’s limit of detection (LOD) was 0.1 µg/dL. Samples with a PbB concentration below LOD were entered into the database with a LOD/2 value (i.e., 0.05 µg/dL). For CdB, quality control samples from IAL were reanalyzed every ten samples. The precision and accuracy obtained were 7.6% and 19.5%, respectively. The LOD was set at 0.01 μg/L, and results below this limit were entered into the dataset as LOD/2. Concerning blood lead levels (PbB), nine women (5.4%) had values below the LOD. On the other hand, 28 women (23.2%) had CdB levels below this limit.

### 2.4. Kidney Function Assessment

Kidney function was analyzed using eGFR, which was determined by serum creatinine concentrations in spot urine sample. Creatinine was measured according to the Jaffe method (Thermo Scientific Konelab, Finland) and the kit manufacturer’s instructions (Wiener Lab, Rosario, Argentina). For those 18 years of age or older, eGFR was calculated using the Chronic Kidney Disease Epidemiology Collaboration (CKD-EPI) equation without race-adjustment [31,32]. For those under 18 years of age, the Chronic Kidney Disease in Children (CKiD) equation was used (US Department of Health, National Institute of Diabetes, Digestion, and Kidney Disease).

- CKD-EPI [32]

eGFR (mL/min/1.73 m^2^) = 141 × *min* (CreaS/0.7; 1)^(−0.241)^ × *max* (CreaS/0.7; 1)^(−1.200)^ × 0.9938^age^ × 1.012

where:

CreaS = Serum creatinine in mg/dL

min(Cr/κ, 1) minimum of Cr/0.7 or 1.0

max(Cr/κ, 1) maximum of Cr/0.7 or 1.0

Age (years)

- CKiD

eGFR (mL/min/1.73 m^2^) = k × Ht/CreaS

where:

CreaS = Serum creatinine in mg/dL

Ht = height in cm

K = 0.413

### 2.5. Data Analysis

Frequencies of sociodemographic, lifestyle, and variables are presented as counts and percentages for eGFR < 90, 90 ≤ eGFR < 120, and eGFR ≥ 120 mL/min/1.73 m^2^ [1,33]. Estimated GFR cut-off points were determined by literature recommendations for healthy pregnant women [1,34]. For toxic metal exposure, the data reported for those categories were geometric mean, SD, median, and intervals. For further analyses, these variables were log_10_ transformed for normalization.

Fisher’s exact test or Pearson’s Chi-squared test were applied appropriately to test proportions of categorical variables across eGFR categories. Continuous variables were evaluated using the Shapiro–Wilk (SW) test, and their means (SD) and median (IQR) were included in the tables. A Kruskal–Wallis (KW) test was applied to determine if medians across eGFR categories were statistically different. Post-hoc pairwise comparisons were performed using the Wilcoxon rank sum test with the Holm–Bonferroni method to account for multiple comparisons. Spearman correlations were also performed.

PbB and CdB concentrations were dichotomized by sample median, CDC standards, and literature recommendations. General population recommendations for PbB from the CDC, Ruckart et al. (2021), and Gilbert et al. (2006), were 5 µg/dL, 3.5 µg/dL, and 2 µg/dL, respectively [35,36]. For CdB, the CDC, Kira et al. (2016), and Schulz et al. (2011) recommendations were 0.4 µg/L, 0.6 µg/L, and 1.0 µg/L, respectively [37,38].

Multivariable linear regression analysis was used to estimate the association between log_10_ transformed PbB and eGFR. Prior to adjusting the model, interaction effects were evaluated by ANOVA at *p* < 0.05. Confounders to include in the MLR model were determined by the 10% rule at alpha = 0.05, but due to a limited sample size, we encountered overadjustment bias, and age was the only confounder included and was kept continuous (Supplemental Figure S1). We found interacting effects from alcohol use, CdB tertiles, and contraceptive use and stratified our adjusted model accordingly. Multivariable logistic regression was used to estimate the association between log_10_ transformed PbB and eGFR < 90 mL/min/1.73 m^2^ with the same confounders and stratification as the linear model. All analyses were performed using R-Studio version 4.3.0; significance was chosen at *p* < 0.05 (R Core Team, 2023). We chose to discuss results that were not significant at the chosen alpha but had the magnitude of effect in the anticipated direction and was suggestive of an association with a larger sample size.

## 3. Results

### 3.1. Sociodemographic Data, Lifestyle, and General Exposures to Toxic Metals

For 136 pregnant women from the DSAN-12 cohort, the average serum creatine was 69.6 ± 37.7 µmol/L and the median was 70 µmol/L (IQR = [60, 80]). The average eGFR was 115.4 ± 20.7 mL/min/1.73 m^2^ and the median was 121.8 (IQR = [106, 127.9]) mL/min/1.73 m^2^, which is considered healthy for pregnant women and the general population. The median [IQR] and geometric mean ± sd for PbB were 0.85 µg/dL [0.45, 1.75] and 0.75 ± 3.4 µg/dL. For CdB, they were 0.55 µg/L [0.08, 0.91] and 0.37 ± 3.9 µg/L. These levels were expected, but there is no safe threshold for PbB or CdB concentrations.

Table 1 and Table 2 show that a greater proportion of pregnant women with a low eGFR significantly corresponded to being married, having a history of oral contraceptive use, higher parity, and having contact over the last 10 years with other xenobiotics. Interestingly, the largest proportion of pregnant women who reported having contact with pesticides over the last 10 years were in the eGFR ≥ 120 group. The median and mean age and PbB levels were also statistically different between eGFR categories. Variables expected to influence kidney function such as hypertension, diabetes, heart disease, BMI, water treatment, passive or active smoking status, renovating their house, and domestic waste burning were not statistically different between eGFR categories.

**Figure 1:**
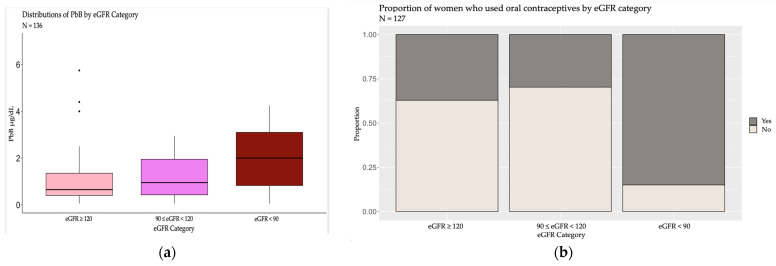
(**a**) Distribution of PbB µg/dL by eGFR category. (**b**) Frequency of contraceptive use by eGFR category.

To identify which eGFR categories were determining the difference, post-hoc comparisons were conducted. Between specific eGFR groups, marital status, parity, contact with pesticides and other xenobiotics over the last 10 years were not statistically significant or the number of observations was <10, and an interpretation could not be reasonably determined. The mean age was significantly higher in 90 ≤ eGFR < 120 compared to the eGFR ≥ 120 group. Figure 1a,b shows the distributions of PbB levels, and the proportion of those that used oral contraceptives before pregnancy were highest in the eGFR < 90 mL/min/1.73 m^2^ group. Age was the highest in 90 ≤ eGFR < 120 mL/min/1.73 m^2^ and statistically different from the eGFR ≥ 120 mL/min/1.73 m^2^ group. Interestingly, age and parity were not statistically different between those who did and did not use oral contraceptive.

Data in Table 3 present the Spearman correlation matrix among kidney function and main continuous covariables. Age was significantly correlated with log_10_ transformed PbB (rho = 0.2, *p* = 0.03), and as age increases, so does Log_10_PbB. Log_10_PbB was moderately inversely correlated with eGFR, as expected, so as Log_10_PbB increases, eGFR decreases (Sp rho = −0.33, *p* = 0.0003). Interestingly, PbB and CdB were not significantly correlated, calling for further investigation.

### 3.2. General Exposures to Pb and Cd

About 93% of the participants had detectable PbB levels, and all women had detectable CdB levels. The proportion of women with PbB and CdB levels above the literature and federal agency’s recommendations by eGFR category are shown in Table 4. Frequencies of eGFR categories were statistically different for PbB above 2 µg/dL [36], PbB above the cohort’s median 0.85 µg/dL, and CdB above the 1.0 µg/L recommendation [38]. Post-hoc comparisons are not in the tables but were performed. They showed that the proportion of women with PbB above the 2 µg/dL recommended level was significantly highest in group eGFR < 90 mL/min/1.73 m^2^ compared to eGFR ≥ 120 mL/min/1.73 m^2^ (*p* < 0.001). It was also higher compared to the 90 ≤ eGFR < 120 mL/min/1.73 m^2^ group, which was expected, although it was not significant. Also, not significant at alpha but suggestive of the expected relationship was the proportion of women with PbB above the median, which was highest in the eGFR < 90 mL/min/1.73 m^2^ group compared to the eGFR ≥ 120 mL/min/1.73 m^2^ group (*p* < 0.082). The proportion of women with CdB levels above the 1 µg/L recommendation in the eGFR < 90 mL/min/1.73 m^2^ group was higher compared to the 90 ≤ eGFR < 120 mL/min/1.73 m^2^ group (*p* < 0.1) [38]. Although, CdB was not higher in the eGFR < 90 mL/min/1.73 m^2^ group compared to the eGFR ≥ 120 mL/min/1.73 m^2^ group, which was unexpected.

Table 5 summarizes the multivariable linear regression analyses for kidney function, continuous eGFR, as the dependent variable. Factors impacting kidney function and potentially eGFR were evaluated for interaction effects and confounding. The interaction terms found included consuming alcohol while pregnant, history of oral contraceptive use, and CdB in tertiles. CdB nor log_10_CdB, log_10_PbB, or eGFR were not analyzed in our study due to sample size limitations and because our analysis was restricted to CdB as an interaction. Our final model adjusted for age and the association between log_10_PbB and eGFR was β = −4.05 (95% CI = [−8.04, 0.05]). Every 1% increase in PbB levels was associated with a 0.02 mL/min/1.73 m^2^ decrease of eGFR. Upon stratification by CdB tertiles, the women with CdB levels in the highest tertile had an association between a log_10_PbB and eGFR of β = −13.90 (95% CI = [−20.83, −6.97]). This trend demonstrates that for those who had the highest CdB levels, every 1% increase in PbB levels was significantly associated with a 0.06 mL/min/1.73 m^2^ decrease of eGFR. Women co-exposed to high levels of CdB and PbB had a decrease in eGFR magnitude 33% higher than if they were exposed to PbB alone. When we stratified by alcohol consumption during pregnancy and previous use of oral contraceptives, there was no significant relationship between log_10_PbB and eGFR.

**Figure 2:**
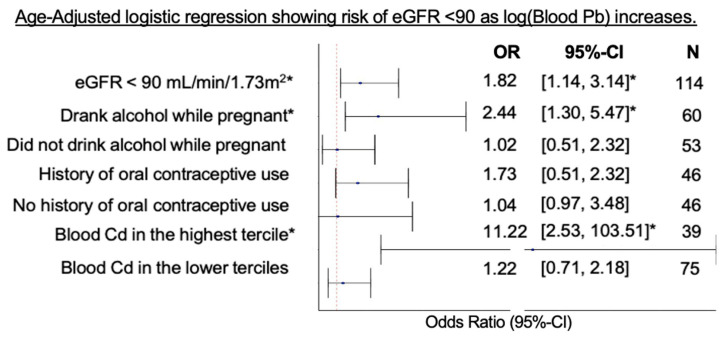
Plot of age-adjusted multivariable logistic regression between Log_10_PbB and eGFR < 90 mL/min/1.73 m^2^ * significant at *p* < 0.05.

Table 6 and Figure 2 summarize the age-adjusted multivariable logistic regression for having eGFR < 90 mL/min/1.73 m^2^ after stratification by effect modifiers. Every 10-fold increase in PbB levels was associated with a 82% higher likelihood of eGFR < 90 mL/min/1.73 m^2^ (OR = 1.82, 95% CI = [1.14–3.14]). However, the participants with elevated PbB levels who reported consuming alcohol during pregnancy were 2.44 times more likely to have reduced eGFR (OR = 2.44, 95% CI = [1.30–5.47]). Moreover, for those with CdB in the highest tertile, the risk of decreased eGFR was much higher when they also had elevated PbB (OR = 11.22, 95% CI = [2.53–103.51]). Since stratified analyses limited the sample size and gave to wider confidence intervals, the following observation was not significant at alpha = 5%, but we found that there is a 73% higher likelihood of decreased eGFR with elevated PbB levels if they have a history of oral contraceptive use (OR = 1.73, 95% CI = [0.97–3.48]).

## 4. Discussion

In this cross-sectional analysis, we identified significant associations between low-level toxic metal exposures and kidney function in pregnant women during their second trimester. Higher PbB concentrations resulted in decreased eGFR, which was more dramatic among those with higher CdB levels. Similarly, during our multivariable logistic regression, we found a significant association for increased odds of eGFR below 90 mL/min/1.73 m^2^ as PbB levels increased among pregnant women who had CdB in the highest tertile compared to those with lower CdB levels. Furthermore, the women who reported drinking alcohol while pregnant also had increased odds of eGFR below 90 mL/min/1.73 m^2^ as PbB levels increased compared to those who did not report drinking alcohol. For the stratified analyses, the sample size was low, leading to wide confidence intervals. Despite that, our adjusted logistic and linear regression models also suggested decreased eGFR as PbB levels increased among women with a history of oral contraceptive use.

Consistent with the literature, our primary finding was the significant inverse association with the eGFR and PbB concentration. CdB was not independently associated with decreased eGFR, which contrasts the literature. For example, in the Park et al. (2022) observational study, they found an elevated risk of decreased eGFR with increased concentrations of Pb, Cd, mercury (Hg), and nickel (Ni) among their South Korean cohort [33]. Their sample was a pull of 1, 984 adults from all genders, 19 years old or older, with an available biospecimen, who participated in Korea’s National Health and Nutrition Survey. Also, their correlation between blood Pb concentration and eGFR reported was comparable to ours in magnitude and significance. Similarly, Kuraeiad and Kotepui (2021) performed a meta-analysis of cross-sectional, longitudinal, or cohort studies reporting on middle-aged adults with occupational-related or environmental risk to lead exposure, blood lead levels, and renal function outcomes [39]. Of the 43 studies analyzed, the authors found that high, medium, and low (<20 µg/dL) PbB levels were associated with abnormal renal function parameters, including blood urea nitrogen (BUN), creatinine, uric acid, and creatinine clearance. In a prospective study of 259 premenopausal women who menstruate regularly, Pollack et al. 2014 also found that each 2-fold increase in the PbB concentration was associated with decreased eGFR regardless of smoking status [34]. Their sample was of women 18–44 years old living around Buffalo, New York, USA. Contrasting our results, Kwon et al. (2023) found an association with CdB and decreased eGFR, but not PbB, in their low-exposure group compared to their reference group [40]. The authors examined the relationship of lead exposure on renal function in 298 individuals, 70.3 years old on average. The participants were recruited because they live near abandoned metal mines and refineries in South Korea and have a moderate-to-high risk of lead exposure. Also, the authors found Urinary N-acetyl-β-glucosaminidase (NAG), another biomarker of kidney dysfunction, was associated with CdB, but not PbB, in both groups. Martinez et al. (2022) examined renal function in 88 adult villagers aged 17–55 years from Simões Filho, Bahia, Brazil, the same region in Brazil as our DSAN-12 cohort [41]. Notably, the eGFR for their women was higher than our cohort’s, even though their cohort was older and non-pregnant, but they did not find an association between eGFR and PbB nor CdB. Also contrasting our results, Martinez et al. (2022) and Park et al. (2022) found an association between PbB and CdB concentrations [33,41].

Although we did not find a correlation between PbB and CdB levels, an interesting result from our analysis was the higher risk of decreased eGFR with increased PbB and CdB in the highest tertile. The relationship between our variables is comparable with the literature, but the magnitude of risk is 10-fold greater than Jain (2019) and Navas-Acien et al. (2009) [42,43]. Jain (2019) performed an analysis like ours stratified PbB and CdB into high and low, and calculated adjusted odds ratios for combinations [42]. The odds ratio for decreased eGFR was twice as risky for the high PbB with high CdB compared to their low PbB and high CdB category. The magnitude and significance of their ORs were comparable to Navas-Acien et al.’s (2009) joint analysis of PbB and CdB levels on reduced eGFR [43]. Both Navas-Acien et al. (2009) and Jain (2019) were using the US NHANES cohort data and were able to adjust for more sociodemographic variables in their linear and logistic regression models [42,43]. Chen et al. (2019) measured PbB and CdB in a Chinese cohort but only found a significant joint association with a reduction in urinary NAG activities [44]. When they performed a similar analysis with urinary Pb and Cd, they found a significant joint association with decreased eGFR. In a Taiwanese cohort, Tsai et al. (2017) examined the joint association with urinary Pb, Cd, and chromium on kidney function [45]. For those in the highest tertile of Cd, the eGFR decreased of comparable magnitude to ours as Pb and chromium doubled.

Our results were consistent with the very limited literature examining how low-level exposure to Pb and Cd affected kidney function in pregnant women. The study that mostly relates to ours was performed by Lin et al. 2023, with predominantly white, high income, pregnant women in eastern Massachusetts [17]. They also calculated eGFR without race adjustment but measured creatinine from blood plasma instead of serum. Lin et al. (2023) adjusted for age, education level, household income, and pregnancy smoking status and found higher PbB was significantly associated with lower eGFR but CdB was not [17]. They also observed an inverse association between a non-essential trace element mixture and eGFR, although their mixture contained PbB, CdB, mercury, arsenic, and cesium. Unlike our study or Lin et al. (2023), Wibowo et al. (2014) performed a case-control study of Indonesian women and measured the specific gravity of urinary Pb and Cd, incidence of preeclampsia, and eGFR [17,18]. Unlike our study, they did not find a significant inverse association with Pb and eGFR, but they did between Cd and eGFR. They did not report any joint association of Pb and Cd with eGFR. Unlike our study, Kort et al. (2022) studied a cohort of Surinamese pregnant women and measured the association of PbB and mercury (Hg) with liver and kidney function [16]. Kidney function was evaluated by urea concentration, not with eGFR. They reported a significant negative association between PbB and urea, which contrasts our findings, and larger literature, that PbB decreases kidney function.

Our sample size limited our analysis, but our logistic regression indicated a significant association between increased PbB and decreased eGFR among those who drank alcohol during pregnancy (alpha = 0.05). Contrasting our results, Lee, Cho, and Kim (2021) found that more frequent alcohol consumption in the prospective cohort was associated with a lesser reduction of eGFR over 12 years independently of baseline kidney function and comorbidities among 5729 Korean adults between 40–79 years old [46]. In the Brazilian population, de Sousa et al. (2023) evaluated 90,846 adults 15 years old or older and found that CKD was less prevalent in the group that consumed alcohol [47]. In alignment with our results, Epstein (1997) found that alcohol can enlarge and alter kidney tubules, which is the anatomical region of the nephron prone to Cd toxicity, and disrupt hormonal control mechanisms [48].

Our logistic regression also hinted at an inverse association between eGFR and PbB among those with a history of hormonal oral contraceptive use. Our data collection did not include specific contraceptives used, but the most frequently reported in Brazil are monophasic combined [49]. These hormonal oral contraceptives are primarily made using estrogen and progestin. Cheung and Lafayette et al. (2013) found that progesterone and oral contraceptives can increase eGFR [1]. Although their conclusions contrast our results, the study highlights the influence of salt and protein intake on the relationship between hormones and eGFR in women. This calls for deeper investigation into our cohort’s nutrition. There were no studies found that explored hormonal contraceptive use, toxic metal exposure, and kidney function. However, Atthobari et al. (2007) found that eGFR fell 6.3% in those who started hormonal contraceptives in their PREVEND study cohort. This cohort of 751 women were aged 28 to, at most, 45 years from Groningen, Netherlands [50]. Furthermore, they found that eGFR was not recovered even after stopping contraceptive use. Although Ahmed et al. (2008) investigated estrogen therapy in postmenopausal women, they also found that estrogen-only, progestin-only, or treatment with both was associated with a significant decline of eGFR compared to non-users [51]. This cohort consisted of 5845 women at least 66 years old in Calgary, Canada. These results demonstrate a relationship between hormonal contraceptives and kidney function in women at different stages of their life, even after contraceptives are no longer being taken. During pregnancy, hormonal changes are drastic and taking contraceptives based on estrogen, progestin, or both may have lasting effects on kidney function. The lack of studies exploring the relationships between hormonal contraceptive use, toxic metal exposure, and kidney function highlights the need for further international investigation.

Our primary limitation is that this was a cross-sectional study, so we are unable to conclude on temporality or causation from our results. Limitations in our analysis also included relatively small sample size and incomplete survey data. Surveys were semi-structured and not all the questions were answered, which limited our ability to fully incorporate all covariables and confounders in our adjusted models. Furthermore, we are still processing other kidney function biomarkers and the data collected on diet. On the other hand, our semi-structured surveys were a strength of the study since we were able to capture risk behaviors, including alcohol consumption, sources of toxic metal exposures, both due to lifestyle and living conditions, and use of hormonal oral contraceptives, which are not present in the current literature.

## 5. Conclusions

Low-level co-exposure to toxic metals may impair kidney function in early pregnancy. The physiological transformation pregnant women undergo is taxing on the human body, but the stresses of pregnancy and exposures to toxic metals are increased when considering the social and health inequities experienced in developing countries and disenfranchised communities in developed countries. Furthermore, prenatal and routine toxic metal screening is not systematically employed, which limits data availability to design intervention efforts. Supporting community-engaged efforts to reduce exposure to toxic metals and to increase socioeconomic conditions would reduce the health care burden in Brazil and abroad. Our findings have important implications for kidney health before and during pregnancy, requiring further studies.

## Figures and Tables

**Table 1:** Sociodemographic characteristics of the study population according to the eGFR categories.

Characteristics	Overall, n = 136 ^1^	eGFR ≥ 120, n = 78 ^1^	90 ≤ eGFR < 120, n = 38 ^1^	eGFR < 90, n = 20 ^1^	*p*-Value ^2^
Ethnicity					0.9
Black/Mixed race	128/136 (94%)	74/78 (95%)	35/38 (92%)	19/20 (95%)	
White/Other	8/136 (5.9%)	4/78 (5.1%)	3/38 (7.9%)	1/20 (5.0%)	
Municipality					0.8
Arauípe	56/136 (41%)	34/78 (44%)	15/38 (39%)	7/20 (35%)	
Nazaré	80/136 (59%)	44/78 (56%)	23/38 (61%)	13/20 (65%)	
Marital Status					0.046 *
Married/Stable Union	72/132 (55%)	37/74 (50%)	19/38 (50%)	16/20 (80%)	
Single	60/132 (45%)	37/74 (50%)	19/38 (50%)	4/20 (20%)	
Socioeconomic Status					0.4
Class A–B–C	60/124 (48%)	34/68 (50%)	19/36 (53%)	7/20 (35%)	
Class D–E	64/124 (52%)	34/68 (50%)	17/36 (47%)	13/20 (65%)	
Household Income					0.12
>1 salary	11/113 (9.7%)	5/64 (7.8%)	6/33 (18%)	0/16 (0%)	
≤1 salary	102/113 (90%)	59/64 (92%)	27/33 (82%)	16/16 (100%)	
Receive Financial Assistance from the Government					0.7
	70/116 (60%)	37/65 (57%)	22/34 (65%)	11/17 (65%)	
Education					0.8
≤Elementary school	73/136 (54%)	41/78 (53%)	20/38 (53%)	12/20 (60%)	
≥Secondary school	63/136 (46%)	37/78 (47%)	18/38 (47%)	8/20 (40%)	
Occupation					0.8
Self-employed/other	87/132 (66%)	49/74 (66%)	26/38 (68%)	12/20 (60%)	
Housewife	45/132 (34%)	25/74 (34%)	12/38 (32%)	8/20 (40%)	
Feel supported socially	90/116 (78%)	48/65 (74%)	29/34 (85%)	13/17 (76%)	0.4
Used oral contraceptives	54/127 (43%)	26/70 (37%)	11/37 (30%)	17/20 (85%)	<0.001 *
Number of children including current pregnancy					0.044 *
First parity	20/62 (32%)	15/33 (45%)	2/18 (11%)	3/11 (27%)	
More than one parity	42/62 (68%)	18/33 (55%)	16/18 (89%)	8/11 (73%)	
Age (years)	26.0 (22.0, 31.0)	25.0 (22.0, 29.0)	30.5 (24.6, 35.0)	26.8 (21.2, 31.9)	0.009 *
BMI pre-gestational (kg/m^2^)	25.1 (4.3)	25.1 (4.9)	24.9 (3.6)	25.3 (4.0)	>0.9

^1^ n/N (%); Mean (SD); Median (IQR). ^2^ Fisher’s exact test; Pearson’s Chi-squared test; Kruskal–Wallis rank sum test. * significant at *p* < 0.05;

**Table 2:** General characteristics and exposure markers of the study population according to the eGFR categories.

Characteristics	Overall, n = 136 ^1^	eGFR ≥ 120, n = 78 ^1^	90 ≤ eGFR < 120, n = 38 ^1^	eGFR < 90, n = 20 ^1^	*p*-Value ^2^
Active smoker	2/79 (2.5%)	1/51 (2.0%)	0/22 (0%)	1/6 (17%)	0.2
Passive smoker	32/124 (26%)	18/69 (26%)	8/36 (22%)	6/19 (32%)	0.8
Waste burning	32/127 (25%)	21/70 (30%)	8/37 (22%)	3/20 (15%)	0.4
Housing renovation	18/122 (15%)	12/67 (18%)	3/35 (8.6%)	3/20 (15%)	0.5
Regular menstrual cycles	85/124 (69%)	46/69 (67%)	24/36 (67%)	15/19 (79%)	0.6
Contact over last 10 years:					
Asbestos	5/65 (7.7%)	2/29 (6.9%)	1/18 (5.6%)	2/18 (11%)	0.9
Radiation	1/65 (1.5%)	1/29 (3.4%)	0/18 (0%)	0/18 (0%)	>0.9
Petroleum	23/129 (18%)	17/72 (24%)	4/37 (11%)	2/20 (10%)	0.2
Dust/Powder	89/129 (69%)	52/72 (72%)	24/37 (65%)	13/20 (65%)	0.7
Pesticides	25/129 (19%)	20/72 (28%)	3/37 (8.1%)	2/20 (10%)	0.032 *
Paints	33/129 (26%)	23/72 (32%)	6/37 (16%)	4/20 (20%)	0.2
Solvents	19/129 (15%)	15/72 (21%)	3/37 (8.1%)	1/20 (5.0%)	0.11
Metal vapors	4/127 (3.1%)	2/70 (2.9%)	1/37 (2.7%)	1/20 (5.0%)	0.8
Other toxicants	3/81 (3.7%)	1/51 (2.0%)	0/23 (0%)	2/7 (29%)	0.019 *
Drinking alcohol while pregnant	62/126 (49%)	33/70 (47%)	16/36 (44%)	13/20 (65%)	0.3
Diabetes while pregnant	1/127 (0.8%)	0/71 (0%)	1/36 (2.8%)	0/20 (0%)	0.4
Heart disease while pregnant	1/127 (0.8%)	1/71 (1.4%)	0/36 (0%)	0/20 (0%)	>0.9
Hypertension while pregnant	15/127 (12%)	9/71 (13%)	3/36 (8.3%)	3/20 (15%)	0.7
At-home water treatment	50/125 (40%)	26/68 (38%)	14/37 (38%)	10/20 (50%)	0.6
Gestational age at sample collection (weeks)	18.5 (5.1)	18.3 (5.1)	18.4 (4.8)	19.3 (5.9)	0.8
	18.0 (15.0, 22.20)	18.0 (14.3, 22.0)	18.0 (16.0, 22.0)	18.5 (16.0, 24.0)	0.8
CdB (µg/L)	0.55 (0.08, 0.91)	0.59 (0.11, 0.99)	0.48 (0.05, 0.74)	0.72 (0.28, 1.55)	0.083 ^+^
PbB (µg/dL)	0.85 (0.45, 1.75)	0.65 (0.40, 1.35)	0.95 (0.43, 1.95)	2.00 (0.83, 3.10)	0.009 *

^1^ n/N (%); Mean (SD); Median (IQR). ^2^ Fisher’s exact test; Pearson’s Chi-squared test; Kruskal–Wallis rank sum test. * significant at *p* < 0.05; ^+^ significant at *p* < 0.10.

**Table 3:** Spearman correlation matrix between PbB, CdB, and continuous covariates.

	Age	Gestation (Weeks)	Log_10_CdB	Log_10_PbB	BMI (kg/m^2^)
**Age**					
Rho					
*p*-value					
N					
**Gestation (weeks)**					
Rho	0.00				
*p*-value	0.99				
N	134				
**Log_10_CdB**					
Rho	0.03	−0.11			
*p*-value	0.75	0.21			
N	123	124			
**Log_10_PbB**					
Rho	0.2	0.04	0.13		
*p*-value	0.03 *	0.67	0.18		
N	114	115	115		
**BMI (kg/m^2^)**					
Rho	0.33	−0.05	−0.06	−0.04	
*p*-value	0.0005 **	0.60	0.52	0.70	
N	108	108	103	96	
**eGFR**					
Rho	−0.29	0.02	0.05	−0.33	−0.04
*p*-value	0.0006 *	0.81	0.60	0.0003 **	0.65
N	134	134	123	114	108

* significant at *p* < 0.05; ** significant at *p* < 0.001.

**Table 4:** Frequencies of PbB and CdB levels above the recommendations by eGFR categories.

Biomarker Reference Values	Overall, n = 136	eGFR ≥ 120, n = 78	90 ≤ eGFR < 120, n = 38	eGFR < 90, n = 20	*p*-Value
**PbB: CDC**					0.6
≥5 µg/dL	3/115 (2.6%)	2/65 (3.1%)	0/31 (0%)	1/19 (5.3%)	
<5 µg/dL	112/115 (97%)	63/65 (97%)	31/31 (100%)	18/19 (95%)	
**PbB: Ruckart (2021)**					0.2
≥3.5 µg/dL	6/115 (5.2%)	4/65 (6.2%)	0/31 (0%)	2/19 (11%)	
<3.5 µg/dL	109/115 (95%)	61/65 (94%)	31/31 (100%)	17/19 (89%)	
**PbB: Gilbert (2006)**					<0.001 *
≥2 µg/dL	23/115 (20%)	5/65 (7.7%)	8/31 (26%)	10/19 (53%)	
<2 µg/dL	92/115 (80%)	60/65 (92%)	23/31 (74%)	9/19 (47%)	
**PbB: Sample median**					0.041 *
≥0.85 µg/dL	58/115 (50%)	27/65 (42%)	17/31 (55%)	14/19 (74%)	
<0.85 µg/dL	57/115 (50%)	38/65 (58%)	14/31 (45%)	5/19 (26%)	
**CdB: CDC**					0.3
≥0.4 µg/L	76/124 (61%)	45/70 (64%)	18/35 (51%)	13/19 (68%)	
<0.4 µg/L	48/124 (39%)	25/70 (36%)	17/35 (49%)	6/19 (32%)	
**CdB: Kira (2016)**					0.2
≥0.6 µg/L	58/124 (47%)	35/70 (50%)	12/35 (34%)	11/19 (58%)	
<0.6 µg/L	66/124 (53%)	35/70 (50%)	23/35 (66%)	8/19 (42%)	
**CdB: Schulz (2011)**					0.004 *
≥1.0 µg/L	27/124 (22%)	17/70 (24%)	2/35 (5.7%)	8/19 (42%)	
<1.0 µg/L	97/124 (78%)	53/70 (76%)	33/35 (94%)	11/19 (58%)	
**CdB: Sample median**					0.2
≥0.55 µg/L	62/124 (50%)	38/70 (54%)	13/35 (37%)	11/19 (58%)	
<0.55 µg/L	62/124 (50%)	32/70 (46%)	22/35 (63%)	8/19 (42%)	

* significant at *p* < 0.05.

**Table 5:** Age-adjusted multivariable linear regression analyses for eGFR as a continuous outcome.

	eGFR mL/min/1.73 m^2^	Alcohol Use during Pregnancy	Did not Use Alcohol while Pregnant	History of Oral Contraceptive Use	No History of Oral Contraceptive Use	Highest CdB Tertile	Lower CdB Tertiles
Intercept	114.49 ** [110.59, 118.39]	110.74 ** [104.06, 117.43]	115.49 ** [109.23, 121.74]	107.52 ** [99.90, 115.14]	118.98 ** [115.31, 122.64]	112.51 ** [105.73, 119.29]	115.52 ** [111.02, 120.02]
Log_10_PbB	−4.05 * [−8.04, 0.05]	−1.63 [−8.72, 5.46]	1.93 [−4.42, 8.27]	−7.29 [−15.05, 0.46]	1.01 [−2.88, 4.91]	−13.90 ** [−20.83, −6.97]	0.48 [−4.16, 5.11]
Age	−2.78 [−6.77, 1.22]	−5.00 [−12.09, 2.09]	−3.30 [−9.65, 3.04]	−7.06 [−14.82, 0.70]	−2.29 [−6.19, 1.60]	−3.39 [−10.32, 3.53]	−2.56 [07.20, 2.07]
N	114	42	53	46	67	39	75
R^2^	0.06	0.07	0.03	0.15	0.02	0.34	0.02

** *p* < 0.001; * *p* < 0.05.

**Table 6:** Age-adjusted multivariable logistic regression between Log_10_PbB and eGFR < 90 mL/min/1.73 m^2^ stratified by major determinants.

Predictors	eGFR < 90 mL/min/1.73 m^2^			Alcohol Use during Pregnancy			History of Oral Contraceptive Use			Highest CdB Tertile		
	*OR*	*95% CI*	*p*	*OR*	*95% CI*	*p*	*OR*	*95% CI*	*p*	*OR*	*95% CI*	*p*
Intercept	0.23	0.02–2.45	0.226	0.28	0.01–7.29	0.438	0.09	0.00–1.43	0.096	0.03	0.00–3.82	0.177
Log_10_PbB	1.82	1.14–3.14	0.019 *	2.44	1.30–4.47	0.013 *	1.73	0.97–3.48	0.086	11.22	2.53–103.51	0.009 *
Observations	114			60			46			39		
N	144			113			113			114		

* *p* < 0.05.

## Data Availability

The raw data supporting the conclusions of this article will be made available by the authors upon request.

## Supplementary Materials

The following supporting information can be downloaded at: https://www.mdpi.com/article/10.3390/toxics12040261/s1, Figure S1: DAG for regression models.
