# Supplementary figures and images for: A Cross-Sectional Analysis Investigating Pregnant Women’s Renal Function and Its Association with Lead and Cadmium Exposures—The DSAN Birth Cohort Study in Recôncavo Baiano, Brazil
Source: Toxics. 2024 Mar 30;12(4):261. doi: 10.3390/toxics12040261 (PMC11054989; doi:10.3390/toxics12040261)

**Supplemental Figure S1.** DAG for regression models

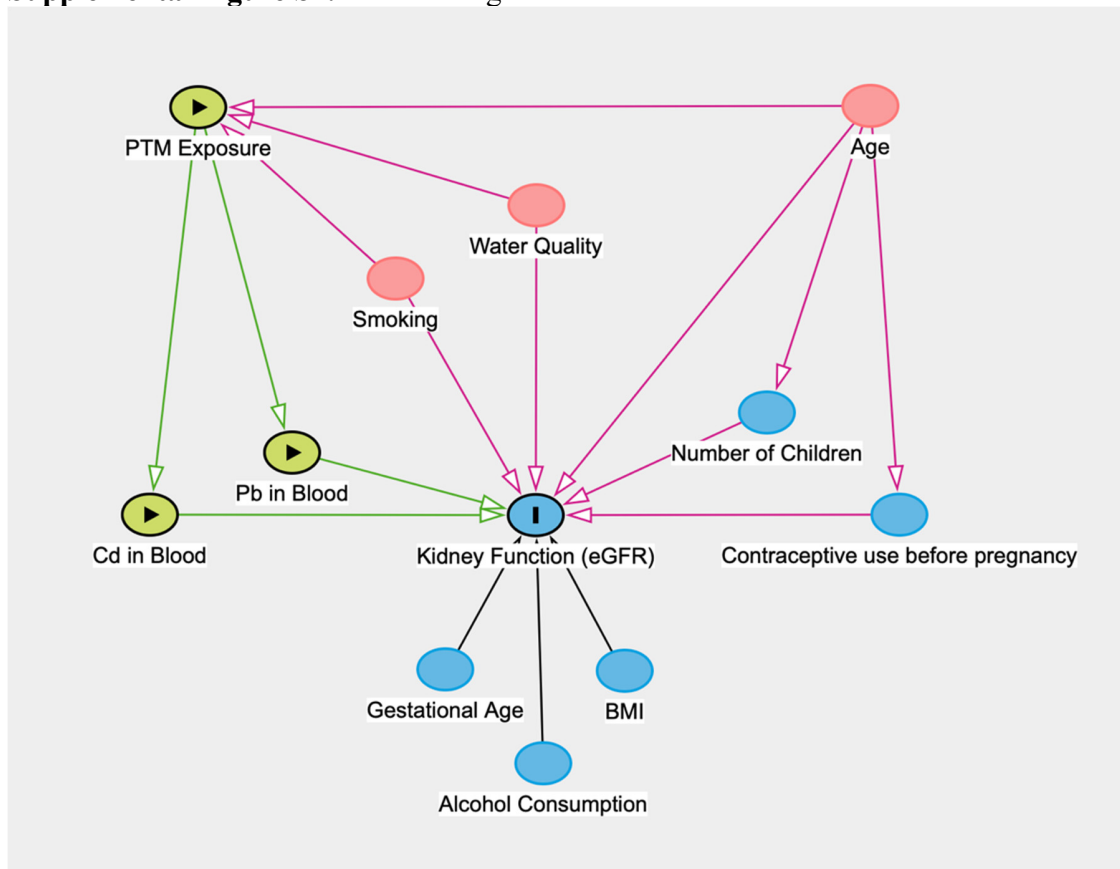

Supplement: Supplementary file 1 [file toxics-12-00261-s001.zip › toxics-2937565-supplementary.pdf]
